# Nonlinear Association Between BMI and Depression Among Nonalcoholic Fatty Liver Disease: NHANES (2017–2018)

**DOI:** 10.1155/bn/8868665

**Published:** 2025-07-28

**Authors:** Hui Peng, Dan Zhou, Yuan Dai, Weifeng Chen

**Affiliations:** General Medicine Department, Shenzhen Second People's Hospital (The First Affiliated of Shenzhen University), Shenzhen, Guangdong, China

**Keywords:** body mass index, depression, nonalcoholic fatty liver disease, nonlinearity

## Abstract

**Background:** Depression is one of the most common diseases in the world. Earlier research on the link between body mass index (BMI) and depression has been contentious. This study seeks to investigate the connection between BMI and depression among individuals with nonalcoholic fatty liver disease (NAFLD).

**Methods:** All data were extracted from the National Health and Nutrition Examination Survey (NHANES) database 2017–2018. The Cox regression technique was employed to analyze the link between BMI and depression. To analyze the potential nonlinear connection between BMI and depression, Cox proportional hazards regression incorporating cubic spline functions and smooth curve fitting was utilized. In addition, a two-segment Cox proportional hazards regression model was used to pinpoint the inflection point at which BMI impacts the likelihood of depression. The Patient Health Questionnaire (PHQ-9) was the primary measure of depressive symptoms.

**Results:** The mean age of the 1426 participants was 56.05 years with a standard deviation of 15.63 years, and approximately 49.30% of the sample were male. After controlling for confounding variables, BMI demonstrated a positive association with depression (OR:1.05, 95% CI:1.02–1.09, *p* < 0.004). The two-piecewise Cox proportional hazards regression model identified an inflection point for BMI at 29.8 kg/m^2^. Below this inflection point (BMI ≤ 29.8 kg/m^2^), BMI was positively correlated with an increased risk of depression (OR:1.23, 95% CI:1.04–1.45, *p* < 0.014). Conversely, when BMI exceeded 29.8 kg/m^2^, the association was not statistically significant (OR: 1.02, 95% CI: 0.98–1.07, *p* = 0.305).

**Conclusion:** There is a nonlinear relationship between BMI and depression among patients with NAFLD. BMI was positively related to depression when BMI is less than 29.8 kg/m^2^.

## 1. Introduction

NAFLD is among the most prevalent liver diseases globally and is intricately linked to metabolic disorders such as obesity, diabetes, and cardiovascular diseases [1]. As a chronic liver condition with the potential to cause severe complications, the global adult prevalence of NAFLD was approximately 37% as of 2019, presenting a substantial public health challenge [2]. Concurrently, depression represents another major public health concern. Between 2013 and 2016, 8.1% of American adults aged 20 and over experienced depression within any given 2-week period, with approximately 80% of these individuals reporting at least some impairment in work, home, and social activities due to their condition [3]. Depression not only diminishes individual quality of life but also imposes significant economic and social burdens on society. In Europe, it accounts for 5%–10% of the disease burden, while in the United States, the annual economic impact exceeds $210 billion [4].

Depression is a multifaceted condition resulting from the interplay of social, psychological, and biological factors, and it is recognized to have a bidirectional relationship with various clinical diseases, including obesity, hypertension, Type 2 diabetes mellitus (T2DM), and cardiovascular diseases [5–7]. Current research indicates a significant bidirectional association between NAFLD and depression [8, 9]. Notably, the prevalence of depression among individuals with NAFLD is approximately 18.21%, markedly higher than that observed in the non-NAFLD population [10]. Furthermore, the 10-year incidence rate of depression in NAFLD patients was found to be 21.2%, compared to 18.2% in non-NAFLD individuals [11].NAFLD and depression share several risk factors, such as obesity, metabolic syndrome, and T2DM [12]. In the context of NAFLD, dysregulated lipid metabolism in adipose tissue is closely linked to the pathogenesis of depression, underscoring the critical role of obesity and adipose tissue function within the liver-brain axis [13].

BMI is a widely utilized metric for assessing obesity and increased adiposity, and it is acknowledged as a risk factor for NAFLD [10, 14]. Nevertheless, substantial discrepancies persist in the literature concerning the association between BMI and depression. Certain studies have identified positive linear correlations [15–17].whereas others have proposed the presence of a U-shaped or negative correlation [18]. These variations may be attributable to population-specific factors such as gender, metabolic health status, or comorbidities. While depression has been individually linked to both BMI and NAFLD, the intricate interactions among these three variables—particularly the nature of the relationship between BMI and depression within the NAFLD population—remain underexplored. Current research predominantly focuses on the linear relationship between BMI and depression in individuals with NAFLD [19, 20], lacking a comprehensive investigation into potential nonlinear dynamics or threshold effects.

Drawing on the metabolic and inflammatory dysregulation characteristics associated with both NAFLD and depression, we propose an innovative hypothesis: within the context of NAFLD, the relationship between BMI and depression may exhibit a nonlinear piecewise pattern rather than adhering to a conventional linear trajectory. To further investigate this relationship, we employed data from the NHANES in the United States with the following objectives: (1) conducting an in-depth analysis of the morphological characteristics of the BMI-depression relationship and (2) identifying potential inflection points of BMI and examining its moderating effect on the association with depression. By moving beyond the traditional linear hypothesis, this study not only contributes to the existing body of literature but also has the potential to inform more precise and personalized clinical intervention strategies for NAFLD patients across different BMI categories.

## 2. Methods

### 2.1. Study Design and Participants

The National Health and Nutrition Examination Survey (NHANES) is a series of multistage cross-sectional surveys conducted nationwide in the United States since 1959. The data included demographics (age, sex, ethnicity, etc.), dietary intake, examinations (blood pressure, body mass index (BMI), ultrasound examination of the liver, etc.), biochemical measures (liver and kidney function, etc.), and information collected by questionnaires (disease history, medications, psychology, etc.). The NHANES protocol was approved by the Research Ethics Review Board and the National Center for Health Statistics, and all subjects signed informed consent forms. We extracted data from NHANES 2017–2018. A total of 9254 NHANES patients from 2017 to 2018 were included in this study. To ensure the validity and accuracy of the study, we excluded participants who included pregnant women, adolescents younger than 20 years of age, BMI greater than 50 kg/m^2^, and participants who did not fully complete the liver ultrasound transient elastography and depression questionnaires. In the end, the number of participants included in the study was 1426.

### 2.2. Variables

#### 2.2.1. Nonalcoholic Fatty Liver Disease (NAFLD)

**Figure 1:**
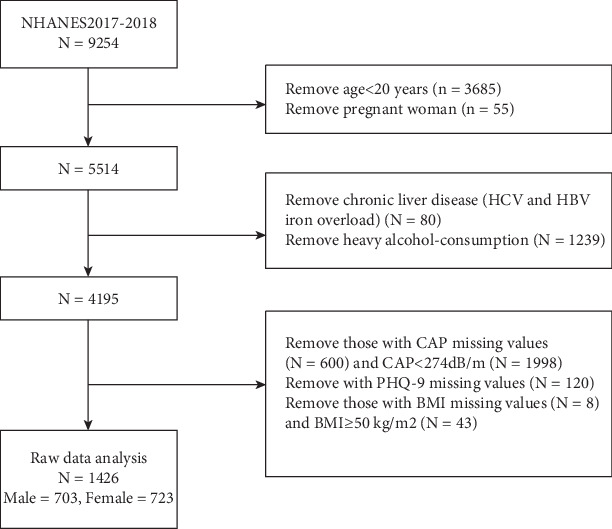
Flow diagram of subjects included in the study.

Vibration controlled transient elastography (VCTE) was conducted during the 2017–2018 cycle using the FibroScan Model 502 V2 Touch (Echosens, Paris, France). The controlled attenuation parameter (CAP) and liver stiffness measurement (LSM) of VCTE are noninvasive methods that are capable of simultaneously measuring liver steatosis and fibrosis [21]. The examination was administered by NHANES technicians following a comprehensive 2-day training program under the guidance of an expert technician. Machines initially used the M probe unless the XL probe was indicated by them. The interrater reliability was assessed between health technicians and expert FibroScan technicians on a sample of 32 subjects, resulting in a reported stiffness reliability coefficient of 0.86 (with a mean difference of 0.44 ± 1.3 KPa) and a CAP reliability coefficient of 0.94 (with a mean difference of 4.5 ± 19.8 db/m). Prior to the examination, the participants were instructed to fast for at least 3 h prior to measurements and to lie in a supine position with their right arm fully abducted. A right liver lobe scan was conducted using an intercostal approach. In this study, CAP and LSM values were expressed in decibels per meter and kilopascals, respectively. Steatosis in NAFLD participants was defined as a CAP value of ≥ 274 dB/m, as established by a recent influential study conducted by Eddowes et al. [22]. Subjects were defined as NAFLD if their CAP value was ≥ 274 dB/m in the absence of (1) positive Hepatitis B surface antigen or positive HCV RNA and (2) excessive overconsumption of heavy alcohol (more than two alcoholic drinks a day for men, 1 alcoholic drink for women) [23] (Figure 1).

#### 2.2.2. Depression

The assessment of depressive symptoms over the past 2 weeks was conducted using the PHQ-9, a 9-item screening tool. The items were scored from 0 to 3, with zero indicating no symptoms, 1 indicating a few days of symptoms, 2 indicating more than half of the days of symptoms, and 3 indicating almost daily symptoms. There was a range of 0 to 27 on the PHQ-9. According to the aforementioned study, participants who scored 10 or higher on the PHQ-9 were classified as having depressive symptoms [24].

#### 2.2.3. Other Factors

##### 2.2.3.1. BMI

BMI was calculated by dividing weight in kilograms by height in meters squared.

##### 2.2.3.2. Hypertension

A mercury sphygmomanometer was used to measure blood pressure, followed by three consecutive auscultatory blood pressure readings after a 5-min rest period. As a representative value for both systolic and diastolic blood pressures, the mean of the three readings was used. The physician's clinical judgment, the patient's use of antihypertensive medication, and the average arterial systolic pressure ≥ 140 mmHg or diastolic pressure ≥ 90 mmHg were also considered.

##### 2.2.3.3. Diabetes

Diabetes Type 2 was diagnosed if participants had a previous diagnosis or used antidiabetic drugs or if their hemoglobin A1c (HbA1c) level was at least 6.5% or when their blood glucose level was 126 mg/dL or higher during the survey. For Borderline, participants were considered to have the condition if they reported a prediabetes or if they had a hemoglobin HbA1c 5.7%–6.4%or a blood glucose level of 100–125 mg/dL.

##### 2.2.3.4. Smoking

In this study, “smoking” refers to smoking at least 100 cigarettes in a lifetime, then dividing that into no (< 100 or never smoking) and yes (> 100).

Among the participants, age, gender, race (categorized as Mexican American, other Hispanic, non-Hispanic White, non-Hispanic Black, and other race—including multiracial), family income to poverty ratio, education level, physical activity, chronic liver disease, and previous medical history were self-reported.

### 2.3. Statistical Analysis

An analysis of the data was conducted using EmpowerStats (https://www.empowerstats.net, X&Y solutions, Inc. Boston, Massachusetts). A *p* value of 0.05 was considered statistically significant. Statistics were used to describe the baselines for the study population by BMI subgroup. Continuous variables were described by means ± standard deviation and weighted linear regression models. We calculated beta values and 95% confidence intervals (CIs) between depression and BMI using multivariate linear regression analysis. Three models were used to build the multivariate test: Model 1: No variables were adjusted; Model 2: adjusted for gender, age, and race; and Model 3: adjusted for all the possible covariables. In order to examine the relationship between depression and BMI, smoothed curve fits were carried out simultaneously with threshold effects analysis. An advanced method of dealing with missing values is multiple imputation (MI).

## 3. Results

### 3.1. Baseline Characteristics

The participants were divided into three tertiles based on their BMI values: T1 (18.3–28.8 kg/m^2^), T2 (28.9–34 kg/m^2^), and T3 (34.1–49.9 kg/m^2^) (Table 1). There was a negative correlation between BMI and age, as well as BMI and income. Additionally, BMI showed positive correlations with diabetes and hypertension. Significant disparities were observed in the distribution of gender, race, education, and physical activity across the BMI tertiles. Moreover, a positive correlation between BMI and depression was evident, with the percentage of participants with depression increasing from T1 to T3 (3.85%, 9.17%, and 12.11%, respectively).

### 3.2. Univariate Analysis of Depression

The results of univariate analysis are presented in Table 2. Univariate linear regression showed that age, race, income, education, marital status, physical activity, and hypertension were not associated with depression. Furthermore, univariate analysis showed that BMI (OR = 1.07, 95%CI = 1.04–1.10, *p* < 0.001), gender (OR = 1.83, 95%CI = 1.24–2.70, *p* = 0.002), diabetes (OR = 1.70, 95% CI: 1.04– 2.75, *p* = 0.033), and smoking (OR = 1.50, 95%CI = 1.03–2.18, *p* = 0.036) were positively correlated with depression.

### 3.3. Association Between BMI and Depression

Multiple logistic regression models were constructed to explore the association between BMI and depression (Table 3). In model 1, no variables were adjusted. Compared to the T1 group, high BMI levels (T2 and T3 group) had significantly positive associations with depression (OR = 2.52, 95%Cl = 1.43–4.42, and OR = 3.44, 95%Cl = 1.99–5.93, *p* for trend < 0.001). In Model 2, age, gender, and race were further adjusted. Compared to the referent group, high BMI levels (T2 and T3 group) remained significantly positive associations with depression (OR = 2.59, 95%CI = 1.46–4.60 and OR = 3.47, 95%CI = 1.95–6.17, *p* for trend < 0.001). To further exclude the influence of covariables, gender, age, race, family PIR, hypertension, diabetes, smoking, marital status, physical activity, and education level were adjusted in Model 3. The highest BMI level still had a significant positive association with depression (OR = 2.95, 95%CI = 1.54–5.66, *p* for trend 0.03).

### 3.4. The Analyses of Nonlinear Relationship

**Figure 2:**
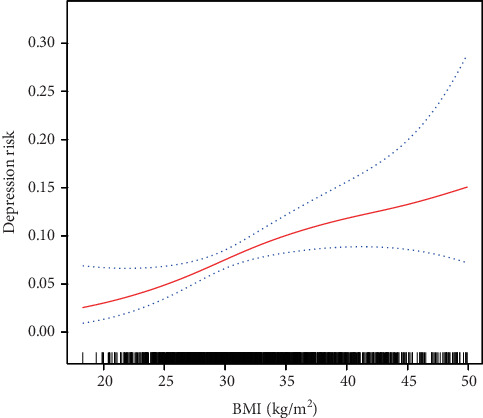
Association between BMI and depression. A threshold and nonlinear association between BMI and depression was found (*p* < 0.05) using a generalized additive model (GAM) after adjusting for gender, age, race, family PIR, hypertension, diabetes, smoking, marital status, physical activity, and education level. The smooth curve fitting the variables is shown by a solid red line. The blue bands indicate the 95% confidence interval of the fit.

A generalized additive model curve analysis was used to evaluate the diagnostic capability of BMI for depression (Figure 2). We found that the relationship between BMI and depression was nonlinear (after adjusting age, gender, race, family PIR, hypertension, diabetes, smoking, marital status, physical activity, and education level). By using a two-piecewise linear regression model, we calculated that the inflection point was 29.8 kg/m^2^.On the left side of the inflection point, the incidence increased by 23% per 1 kg/m^2^ in BMI (OR = 1.23; 95%CI = 1.04–1.45; *p* = 0.014) (Table 4). On the right side of the inflection point, the effect size had no statistical significance (OR = 1.02; 95%CI = 0.98–0.07; *p* = 0.3052).

## 4. Discussion

This cross-sectional study examined the association between BMI and depression among patients with NAFLD. Based on the analysis of information from 1426 patients, we observed a nonlinear relationship between BMI and depression, and there was a segmental. On the left side of the inflection point (BMI ≤ 29.8 kg/m^2^), depression increased by 1.23 for each additional unit of BMI (OR = 1.23, 95% CI: 1.04–1.45, *p* = 0.014). On the right side of the inflection point, the obvious relationship could not be observed (OR = 1.02, 95% CI: 0.98–1.07, *p* = 0.305).

Numerous prior studies have investigated the association between BMI and depression, most studies demonstrating that an elevated BMI is linked to an increased risk of depression [16, 17, 25]. Furthermore, research utilizing twin data has corroborated this finding, revealing a positive correlation between higher BMI and the likelihood of experiencing depression [26]. In the context of NAFLD, existing literature indicates a similar positive correlation between BMI and depression among NAFLD patients [19, 20]. Our study further substantiates these findings, showing that, even after adjusting for confounding variables, the risk of depression escalates with an increase in BMI, aligning with the results of previous research. However, empirical research has also demonstrated that extreme BMI values, whether excessively low or high, are correlated with an increase in depressive symptoms, typically exhibiting a U-shaped relationship. This indicates that individuals who are underweight, overweight, or obese are at an elevated risk of experiencing depression [18, 27]. This finding suggests that the association between BMI and depression is intricate and potentially influenced by multiple factors. In this study, through curve fitting analysis, it was further identified that a nonlinear relationship exists between BMI and depression in patients with NAFLD. Specifically, when BMI is below 29.8 kg/m^2^, there is a significant positive correlation with depression; however, this correlation becomes nonsignificant when BMI exceeds 29.8 kg/m^2^. This result underscores the complexity of the BMI-depression relationship in patients with NAFLD and indicates the necessity for further research to elucidate the underlying mechanisms.

The following reasons may explain the relationship between BMI and depression in patients with nonalcoholic conditions. Research has demonstrated that an elevated BMI is commonly linked to chronic low-grade inflammation, with a particular emphasis on the accumulation of visceral adipose tissue. This visceral fat is known to secrete proinflammatory cytokines, which further amplify systemic inflammatory responses. These inflammatory mediators are regarded as pivotal contributors to the pathogenesis of NAFLD [28–30]. In comparison to healthy individuals, patients suffering from depression exhibit significantly higher levels of proinflammatory cytokines [31]. Current studies have substantiated a strong interconnection between inflammation, metabolic processes, and depression, especially within obese populations, where an increase in BMI exacerbates inflammation associated with depression [32, 33]. Concurrently, individuals who are overweight or obese frequently exhibit dysregulation of the hypothalamic–pituitary–adrenal axis, which is integral to cortisol production. Research indicates that obese individuals with depression have significantly elevated serum cortisol levels compared to their nondepressed counterparts, and sustained cortisol elevation can exacerbate depressive symptoms in this demographic [34]. Additional studies have demonstrated that in patients with abdominal obesity, the presence of depression and anxiety is associated with hyperactivity of the HPA axis, resulting in excessive cortisol secretion [35]. This scenario establishes a feedback loop wherein obesity contributes to increased cortisol levels, and heightened cortisol further exacerbates depressive symptoms, thereby worsening the condition [36]. Furthermore, the dysbiosis of the gut microbiota in individuals with obesity is intricately linked to emotional regulation and is posited to play a crucial role in the pathogenesis of depression [37]. Empirical studies have demonstrated significant differences in the microbiota composition between obese and nonobese individuals, suggesting that this dysbiosis may influence host metabolism and neurocognitive functions, thereby exacerbating the incidence of depression and related disorders [38]. Additionally, the association between BMI and depression is significantly modulated by psychosocial factors. Individuals with moderate obesity are often more vulnerable to depression due to experiences of social discrimination and concerns related to body image, which profoundly affect their self-esteem and mental health [39, 40]. Social judgment frequently results in heightened negative self-perception and feelings of worthlessness. Research underscores that negative body image is a pivotal factor in the relationship between obesity and depressive symptoms [40].

This study identified a nonlinear relationship between BMI and depressive symptoms within the NAFLD population, with a turning point at a BMI of 29.8 kg/m^2^. This relationship may be attributed to the distinct metabolic disorder characteristics inherent to NAFLD patients. For individuals with a BMI less than or equal to 29.8 kg/m^2^, each incremental increase of one unit in BMI is associated with a 23% heightened risk of depression. This finding likely reflects the combined effects of metabolic abnormalities, such as chronic inflammation, alongside psychological stressors, including body image concerns and social discrimination, particularly in moderately obese NAFLD patients. Conversely, beyond the turning point, this association is no longer evident (odds ratio = 1.02, *p* = 0.305), indicating that severely obese NAFLD patients may possess mechanisms that mitigate the risk relationship between BMI and depression. These mechanisms may include the following: (1) the earlier implementation of metabolic syndrome management strategies, such as glycemic and lipid-lowering treatments, which improve inflammatory status [41]; (2) the development of psychological resilience through long-term adaptation to the disease [42, 43]; (3) more frequent follow-up care, which provides psychological support from social and healthcare systems for obese patients; and (4) cognitive function changes due to severe metabolic disorders may mask some depressive symptoms. The nonlinear trend indicates that for the particular NAFLD population, a metabolic compensation threshold could exist in the pathological mechanisms associated with BMI-related depression.

There are some limitations to our study. Firstly, this study is a cross-sectional analysis, so temporality and causality cannot be determined, and a causal relationship between BMI and depressive symptoms cannot be confirmed. Secondly, self-reported questionnaires and subjective reports were used in this study, which can lead to inaccurate results because of recall bias. Thirdly, our study subjects were mainly patients with NAFLD, so our findings may not apply to patients with other diseases. Nevertheless, this study offers one important insight. It is the first to identify a turning point between BMI and depression within the NAFLD population. For NAFLD patients with a BMI ≤ 29.8 kg/m^2^, our findings suggest that routine depression screening, such as the PHQ-9, could be considered for integration into metabolic monitoring. This approach may be complemented by early psychosocial interventions aimed at addressing body image concerns. Additionally, it is essential to evaluate changes in inflammatory markers and gut microbiota. While these targeted interventions may help in addressing the reciprocal risk of metabolic and mental health decline, further longitudinal studies are necessary to validate these suggestions. Future research should also include dynamic changes in BMI, longitudinal assessments of body image scales, such as the Body Shape Questionnaire, and relevant data on the effects of treatments like lipid-lowering and glycemic control on depressive symptoms.

## 5. Conclusion

We found that there is a nonlinear relationship between BMI and depression. BMI is positively related to depression when BMI is less than 29.5 kg/m^2^.

## Figures and Tables

**Table 1:** Baseline characteristics of participants (*N* = 1426).

**BMI tertiles (kg/m** ^**2**^ **)**	**T1 (18.30–28.80)** **N** = 467	**T2 (28.90–34.00)** **N** = 480	**T3 (34.10–49.90)** **N** = 479	**p** ** value**
Age (years)	58.54 ± 15.57	55.95 ± 16.13	53.73 ± 14.85	< 0.001
Family PIR	2.82 ± 1.64	2.77 ± 1.61	2.49 ± 1.52	0.006
Gender				< 0.001
Male	254 (54.39%)	255 (53.12%)	194 (40.50%)	
Female	213 (45.61%)	225 (46.88%)	285 (59.50%)	
Race				< 0.001
Mexican American	54 (11.56%)	86 (17.92%)	77 (16.08%)	
Other Hispanic	44 (9.42%)	51 (10.62%)	32 (6.68%)	
Non-Hispanic White	152 (32.55%)	166 (34.58%)	194 (40.50%)	
Non-Hispanic Black	58 (12.42%)	84 (17.50%)	136 (28.39%)	
Other race—including multiracial	159 (34.05%)	93 (19.38%)	40 (8.35%)	
Diabetes				< 0.001
No	159 (34.05%)	158 (32.92%)	116 (24.22%)	
Yes	128 (27.41%)	148 (30.83%)	191 (39.87%)	
Borderline	180 (38.54%)	174 (36.25%)	172 (35.91%)	
Smoking				0.655
No	302 (64.67%)	297 (61.88%)	300 (62.63%)	
Yes	165 (35.33%)	183 (38.12%)	179 (37.37%)	
Physical activity				< 0.001
No	298 (63.81%)	242 (50.42%)	251 (52.40%)	
Yes	169 (36.19%)	238 (49.58%)	228 (47.60%)	
Hypertension				< 0.001
No	252 (53.96%)	222 (46.25%)	199 (41.54%)	
Yes	215 (46.04%)	258 (53.75%)	280 (58.46%)	
Education levels				< 0.001
Less than 11th grade (includes 12th grade with no diploma)	82 (17.56%)	90 (18.75%)	84 (17.54%)	
High school graduate/GED or equivalent	90 (19.27%)	114 (23.75%)	128 (26.72%)	
Some college or AA degree	133 (28.48%)	157 (32.71%)	175 (36.53%)	
College graduate or above	161 (34.48%)	118 (24.58%)	90 (18.79%)	
Marital status				0.076
Married or living with partner	330 (70.66%)	313 (65.21%)	297 (62.00%)	
Widowed	38 (8.14%)	38 (7.92%)	42 (8.77%)	
Divorced or separated	58 (12.42%)	71 (14.79%)	66 (13.78%)	
Never married	41 (8.78%)	56 (11.67%)	73 (15.24%)	
Depression				< 0.001
No	449 (96.15%)	436 (90.83%)	421 (87.89%)	
Yes	18 (3.85%)	44 (9.17%)	58 (12.11%)	

*Note:* Continuous data are expressed as mean ± SD. Categorical data are expressed as *n* (%). One-way ANOVA and the Kruskal–Wallis test are utilized to assess the differences among continuous variables across various BMI groups, while the Chi-square test is employed to examine the distribution differences of categorical variables. A *p* value of < 0.05 was considered statistically significant.

Abbreviations: BMI, body mass index; Family PIR, family income-to-poverty ratio.

**Table 2:** The results of univariate analysis.

**Exposure**	**Statistics**	**OR (95% CI)**	**p** ** value**
BMI (kg/m^2^)	32.29 ± 6.19	1.07 (1.04, 1.10)	< 0.001
Age(years)	56.05 ± 15.63	1.00 (0.99, 1.01)	0.765
Family PIR	2.69 ± 1.59	0.79 (0.69, 0.91)	0.001
Gender			
Male	703 (49.30%)	Reference	
Female	723 (50.70%)	1.83 (1.24, 2.70)	0.002
Race			
Mexican American	217 (15.22%)	Reference	
Other Hispanic	127 (8.91%)	1.19 (0.57, 2.50)	0.649
Non-Hispanic White	512 (35.90%)	0.91 (0.51, 1.60)	0.737
Non-Hispanic Black	278 (19.50%)	1.12 (0.61, 2.08)	0.716
Other race—including multiracial	292 (20.48%)	0.77 (0.40, 1.47)	0.425
Diabetes			
No	433 (30.36%)	Reference	
Yes	467 (32.75%)	1.70 (1.04, 2.75)	0.033
Borderline	526 (36.89%)	1.29 (0.79, 2.11)	0.316
Smoking			
No	899 (63.04%)	Reference	
Yes	527 (36.96%)	1.50 (1.03, 2.18)	0.036
Physical activity			
No	791 (55.47%)	Reference	
Yes	635 (44.53%)	1.37 (0.94, 1.99)	0.101
Hypertension			
No	673 (47.19%)	Reference	
Yes	753 (52.81%)	1.38 (0.94, 2.02)	0.100
Education levels			
Less than 11th grade (includes 12th grade with no diploma)	256 (17.95%)	Reference	
High school graduate/GED or equivalent	332 (23.28%)	0.67 (0.39, 1.14)	0.140
Some college or AA degree	465 (32.61%)	0.68 (0.41, 1.10)	0.118
College graduate or above	369 (25.88%)	0.34 (0.18, 0.62)	< 0.001
Marital status			
Married or living with partner	940 (65.92%)	Reference	
Widowed	118 (8.27%)	1.27 (0.63, 2.54)	0.505
Divorced or separated	195 (13.67%)	2.59 (1.63, 4.10)	< 0.001
Never married	170 (11.92%)	1.32 (0.74, 2.38)	0.348

*Note:* Continuous data are expressed as mean ± SD. Categorical data are expressed as *n* (%). A *p* value of < 0.05 was considered statistically significant.

Abbreviations: BMI, body mass index; CI, confidence interval; Family PIR, family income-to-poverty ratio; OR, odds ratio.

**Table 3:** Association between BMI and depression.

**OR (95% CI) ** **p** ** value**	**Model 1**	**Model 2**	**Model 3**
BMI (kg/m^2^)	1.07 (1.04, 1.10) < 0.001	1.06 (1.03, 1.10) < 0.001	1.05 (1.02, 1.09) 0.004
BMI tertiles			
T1	Reference	Reference	Reference
T2	2.52 (1.43, 4.42) 0.001	2.59 (1.46, 4.60) 0.001	2.51 (1.32, 4.74) 0.005
T3	3.44 (1.99, 5.93) < 0.001	3.47 (1.95, 6.17) < 0.001	2.95 (1.54, 5.66) 0.001
*p* for trend	< 0.001	< 0.001	0.003

*Note:* Model 1: no variables adjusted; Model 2: adjust for gender, age, and race; Model 3: adjust for gender, age, race, family PIR, hypertension, diabetes, smoking, marital status, physical activity, and education level. A *p* value of < 0.05 was considered statistically significant.

Abbreviations: BMI, body mass index; CI, confidence interval; Family PIR: family income-to-poverty ratio; OR, odds ratio.

**Table 4:** Threshold effect analysis of BMI on depression using two-piecewise linear regression.

	**Depression OR (95% CI)**	**p** ** value**
Fitting by weighted linear regression model	1.05 (1.02, 1.09)	0.004
Fitting by weighted two-piecewise linear regression model		
Inflection point of BMI (kg/m^2^)	29.8	
< 29.8	1.23 (1.04, 1.45)	0.014
≥ 29.8	1.02 (0.98, 1.07)	0.305
Log likelihood ratio test	0.038	

*Note:* Two-segment Cox proportional hazards regression models were utilized to assess the data on each side of the inflection points. A *p* value of < 0.05 was considered statistically significant. Adjustment variables: gender, age, race, family PIR, hypertension, diabetes, smoking, marital status, physical activity, and education level.

Abbreviations: BMI, body mass index; CI, confidence interval; Family PIR, family income-to-poverty ratio; OR, odds ratio.

## Data Availability

The data that support the findings of this study are available in NHANES at https://www.cdc.gov/nchs/nhanes/. These data were derived from the following resources available in the public domain: NHANES (https://wwwn.cdc.gov/nchs/nhanes/default.aspx).

## Nomenclature

BMI: body mass index
NAFLD: nonalcoholic fatty liver disease
NHANES: National Health and Nutrition Examination Survey database
PHQ-9: The Patient Health Questionnaire
T2DM: Type 2 diabetes mellitus
VCTE: vibration controlled transient elastography
CAP: controlled attenuation parameter
LSM: liver stiffness measurement
HbA1c: hemoglobin A1c
CI: confidence intervals
OR: odds ratio
Family PIR: family income-to-poverty ratio
